# Supplementary material for: The three Plasmodium falciparum Aurora-related kinases display distinct temporal and spatial associations with mitotic structures in asexual blood stage parasites and gametocytes
Source: mSphere. 2024 Sep 5;9(9):e00465-24. doi: 10.1128/msphere.00465-24 (PMC11423587; doi:10.1128/msphere.00465-24)
Supplement: Supplemental figures and table — Figures S1-S4 and Table S1. [file msphere.00465-24-s0001.pdf]

Supplemental Material

The three *Plasmodium falciparum* Aurora-related kinases display distinct temporal and spatial associations with mitotic structures in asexual blood stage parasites and gametocytes

Matthias Wyss[1,2*], Basil T. Thommen[1,2*], Jacob Kofler[1,2], Eilidh Carrington[1,2], Nicolas M. B. Brancucci[1,2,†] & Till S. Voss[1,2,†]

[1]Department of Medical Parasitology and Infection Biology, Swiss Tropical and Public Health Institute, 4123 Allschwil, Switzerland.

[2]University of Basel, 4001 Basel, Switzerland.

*contributed equally to this work

[†]corresponding authors: till.voss@swisstph.ch; nicolas.brancucci@swisstph.ch.

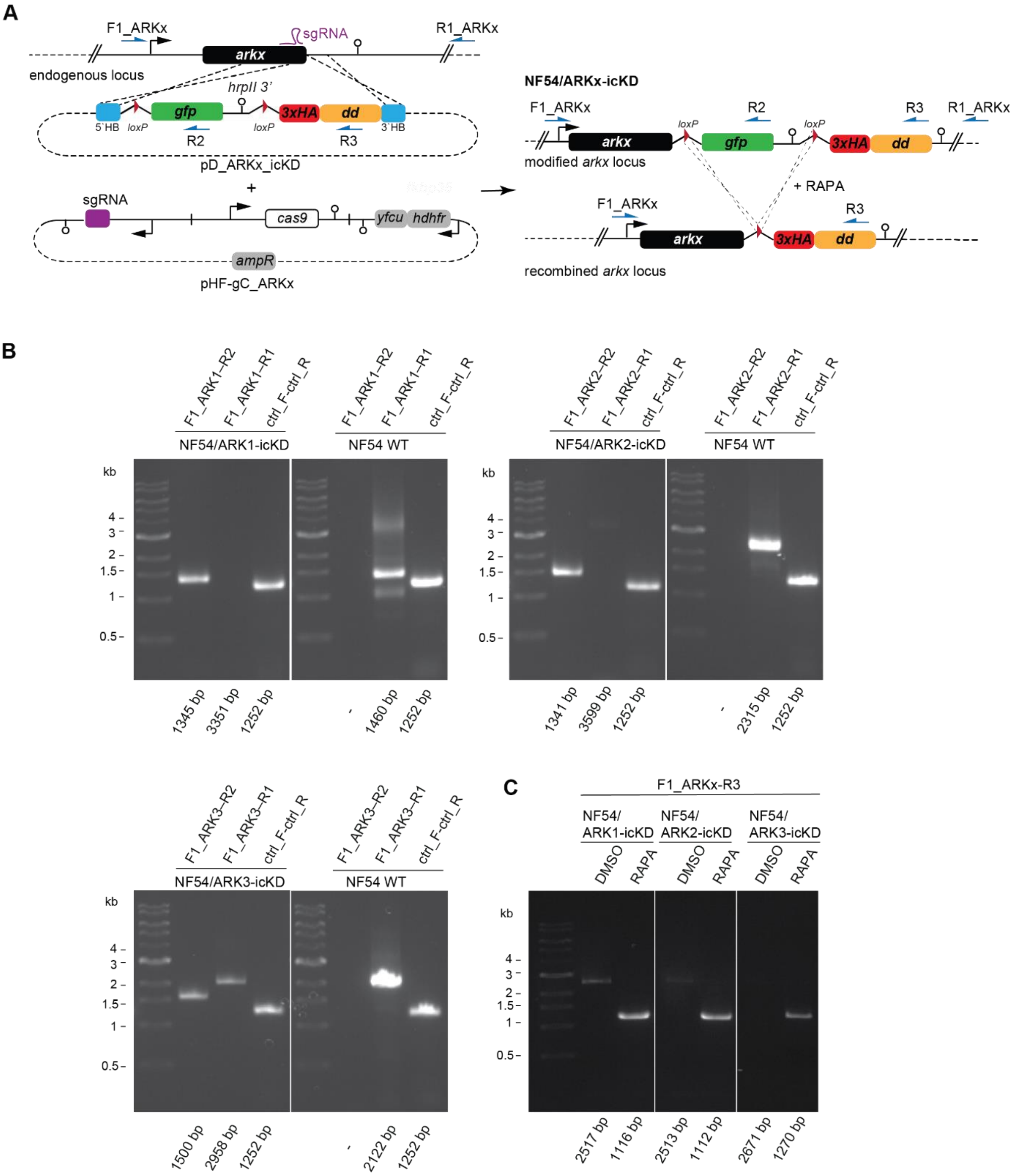

Fig S1. CRISPR/Cas9-based engineering of PfARK1-3 icKD lines. (A) Schematic of the *pfarkx* wild type locus in NF54/DiCre parasites and the plasmids pD_ARKx_icKD and pHF-gC_ARKx (left) that were used to generate the modified *pfarkx* loci in NF54/ARKx-icKD parasite lines (right). Addition of RAPA induces the DiCre-mediated recombination of the two *loxP* sites, thus replacing the C-terminal GFP tag with the 3xHA-DD tag. Primers used for diagnostic PCRs on gDNA are indicated. *x* represents the *pfark* variants 1-3. HB, homology box. *ark*, aurora-related kinase. *gfp*, green fluorescent protein. *3xha*, triple hemagglutinin tag. *dd*, destabilization domain. *yfcu*, yeast cytosine deaminase-uracil phosphoribosyl transferase fusion gene. *hdhfr*, human dihydrofolate reductase. *ampR*, ampicillin

resistance gene. *cas9, S. pyogenes* Cas9. sgRNA, single guide RNA. RAPA, rapamycin. **(B)** Correct locus editing was confirmed by diagnostic PCRs on gDNA of NF54/ARKx-icKD parasites. Numbers at the bottom indicate the expected band sizes. **(C)** Diagnostic PCRs on gDNA of RAPA- and DMSO-treated NF54/ARKx-icKD parasites confirm successful excision of the floxed *gfp* sequence upon RAPA treatment. Numbers at the bottom indicate the expected band sizes.

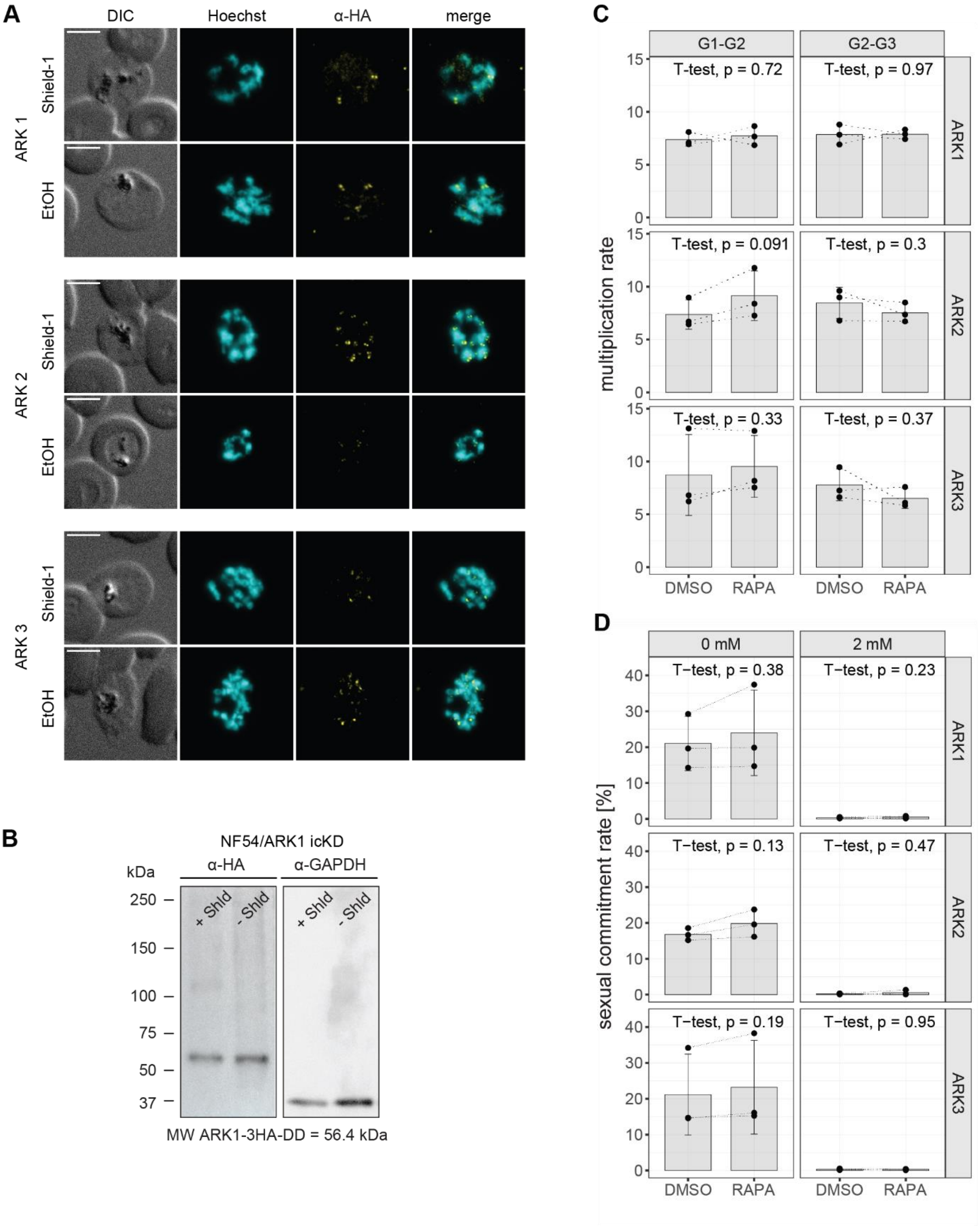

Fig S2. Removal of Shield-1 from RAPA-treated NF54/ARKx-icKD parasites has no effect on parasite muliplication and sexual commitment rates. (A) IFAs of RAPA-treated NF54/ARKx-icKD schizonts expressing 3xHA-DD-tagged PfARKs show that PfARK1-3xHA-DD and PfARK3-3xHA-DD expression is not noticeably reduced in the absence of Shield-1, while PfARK2-3xHA-DD expression is moderately reduced. To induce the knockdown of protein expression, parasites (0-6 hpi) were treated with RAPA for 4 hours, before the population was split and treated with Shield-1 or the

vehicle control EtOH. **(B)** Expression levels of the PfARK1-3xHA-DD fusion protein were assessed by Western blot. Parasite cultures at 0-6 hpi were treated with RAPA for 4 hours, before the population was split and treated with Shield-1 or the vehicle control EtOH. Protein lysates were collected 40 hours later from late schizonts. α-GAPDH antibodies served as loading control. The expected size of the PfARK1-3xHA-DD fusion protein is 56.4 kDa. Expression of the PfARK2-3xHA-DD and PfARK3-3xHA-DD fusion proteins could not be detected by Western blot, most likely due to their low expression levels and large sizes. **(C)** Parasites were split at 0-6 hpi in and treated for 4 hours with either RAPA or DMSO. For both populations, multiplication rates from generation 1 to generation 2 (G1-G2) and G2-G3 were determined by flow cytometry. Data points represent the multiplication rates from G1-G2 and G2-G3 obtained from three biological replicate experiments. Error bars represent the standard deviation. Statistical significance was assessed using a paired two-sided Student's t test. **(D)** Parasites were split at 0-6 hpi in G1 and treated for 4 hours with either RAPA or DMSO and then grown for one replication cycle in mFA in either the presence (2 mM) or absence (0 mM) of choline chloride. Sexual commitment rates were determined based on PfAP2-G-mScarlet expression in the ring stage progeny. Data points represent the proportion of PfAP2-G-mScarlet-expressing parasites among all ring stage parasites obtained from three biological replicate experiments. Error bars represent the standard deviation. Statistical significance was assessed using a paired two-sided Student's t test.

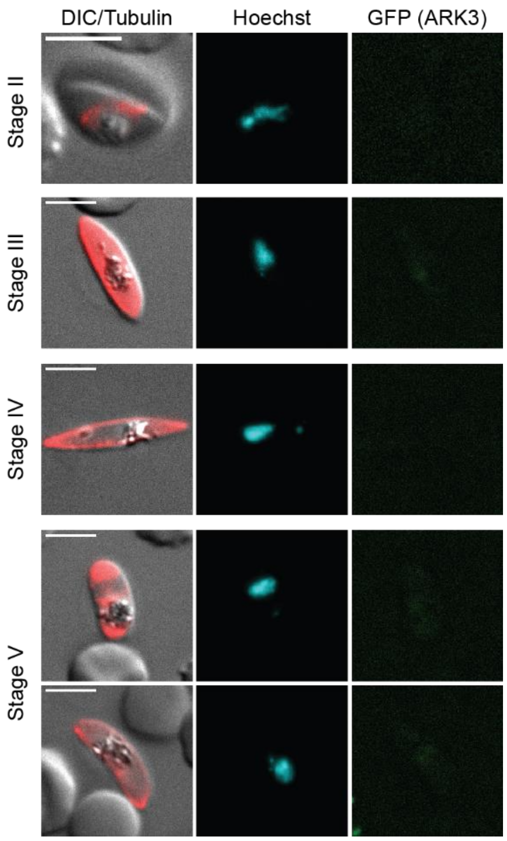

Fig S3. PfARK3 expression was not detected in gametocytes. PfARK3-GFP expression in gametocytes was assessed by live cell fluorescence microscopy. DNA was stained with Hoechst. MTs were stained with SPY555-tubulin. Stages of gametocyte development are indicated. Representative images are shown. DIC, differential interference contrast. Scale bar = 5 µm.

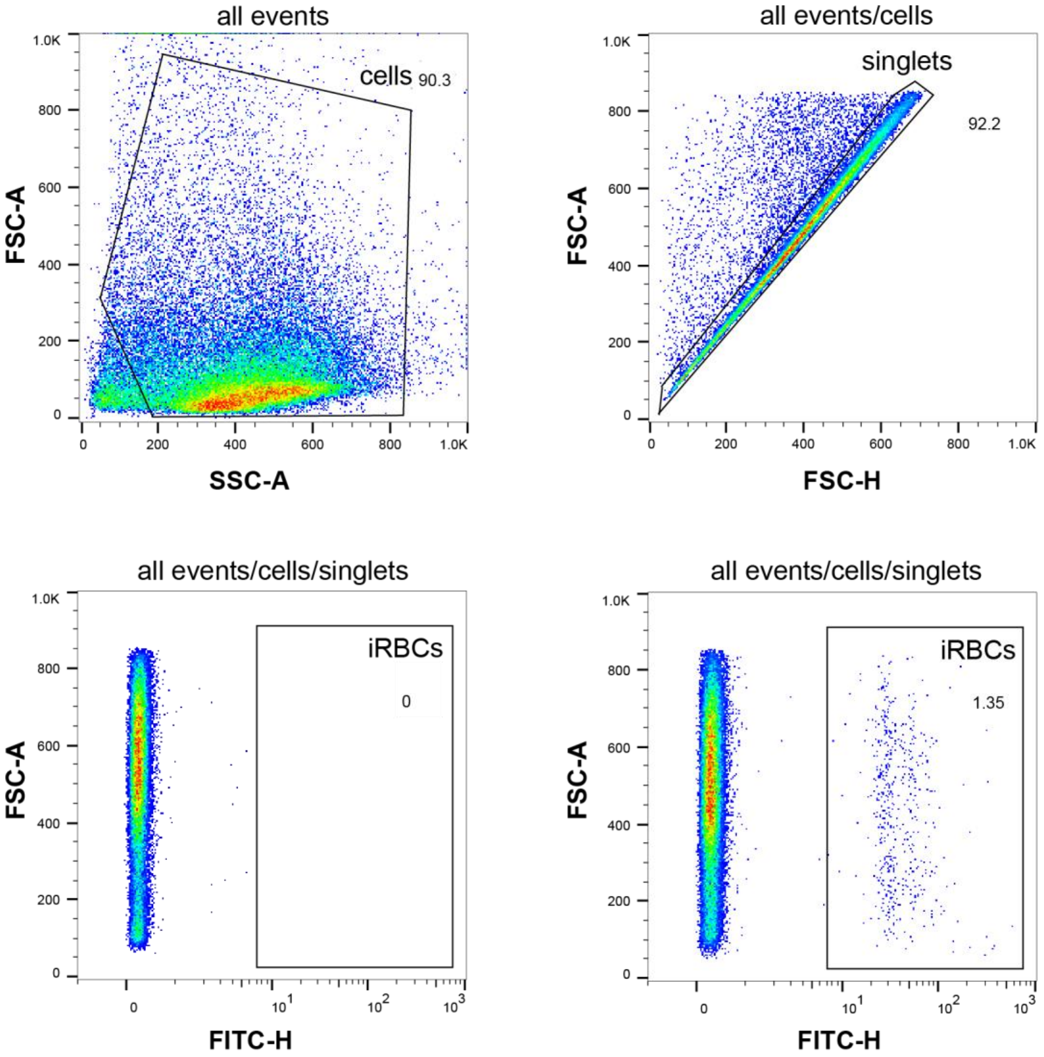

Fig S4. Quantification of parasitaemia by flow cytometry. The gating strategy aimed at distinguishing cells from small debris (FSC-A vs. SSC-A) and removing measurements with more than one cell per droplet (FSC-A vs. FSC-H). Infected red blood cells were identified based on their SYBR Green signal intensity (FSC-A vs. FITC-H). Representative flow cytometry data is shown. The plot at the bottom left was obtained from an unstained control, confirming the specificity of the SYBR Green signal. FSC, forward scatter. SSC, side scatter, FITC, fluorescein isothiocyanate. A, area. H, height.

Table S1: All oligonucleotides used in this study.

name	sequence (5'-3')	used for	cell line/ plasmid
F1_ARK1	ATAATATGGGCTATAAGAACATGTC	PCR on gDNA	NF54/ARK1_icKD
R1_ARK1	CCAAATGGAGGGACAAAATAATG	PCR on gDNA	NF54/ARK1_icKD
F1_ARK2	CGCTCTGAAGGTAATGGC	PCR on gDNA	NF54/ARK2_icKD
R1_ARK2	GTTAATGCTTACATATTCCTCATC	PCR on gDNA	NF54/ARK2_icKD
F1_ARK3	CAACTTTACCTAACATGAATATTGAT	PCR on gDNA	NF54/ARK3_icKD
R1_ARK3	GTAGAATAAATTTGTGAAATACATAAAAG	PCR on gDNA	NF54/ARK3_icKD
R2	GTGTGAGTTATAGTTGTATTCC	PCR on gDNA	NF54/ARKx_icKD
R3	TCATTCCAGTTTTAGAAGCTCCAC	PCR on gDNA	NF54/ARKx_icKD
ctrl_F	AATGGCAGTAACAAAACTTGG	PCR on gDNA	NF54/ARKx_icKD
ctrl_R	CTTAGTTGTTAGTAATGTGTACGG	PCR on gDNA	NF54/ARKx_icKD
PCR1_F	CTGGCGTAATAGCGAAGAGG	cloning	pD_ARKx_icKD
PCR1_R	CATTAATGAATCGGCCAACG	cloning	pD_ARKx_icKD
PCR2_ARK1_F	AGCGAGTCAGTGAGCGAGGAAATAGCACATTTGGATTTGAAACC	cloning	pD_ARK1_icKD
PCR2_ARK1_R	TTTTTTATTTACCTATTTCCCTTATTATGGAACCAAATAGGAGTC	cloning	pD_ARK1_icKD
PCR3_ARK1_F	AGCTTCTAAAACTGGAATGACATATGGTTTCACAACAAATGAC	cloning	pD_ARK1_icKD
PCR3_ARK1_R	CCTTTTCTCTTGTGGATCCGGACATTTGTGACGATTTGTTGG	cloning	pD_ARK1_icKD
PCR4_ARK1_F	CTATTTGGTTCCATAATAAGGGAAATAGGTAAATAAAAAAAATAATATAC	cloning	pD_ARK1_icKD
PCR4_ARK1_R	ATTTGTTGTGAAACCATATGTCATTCCAGTTTTAGAAGCTCC	cloning	pD_ARK1_icKD
PCR2_ARK2_F	AGCGAGTCAGTGAGCGAGGAAGCTAATGGAGGATCAGTACG	cloning	pD_ARK2_icKD
PCR2_ARK2_R	TTTTTTATTTACCTATTTCCAATAAACTGCTTAATCCAAGGATG	cloning	pD_ARK2_icKD
PCR3_ARK2_F	AGCTTCTAAAACTGGAATGAAAAACAATTCATATAAAAGAAAGCG	cloning	pD_ARK2_icKD
PCR3_ARK2_R	CCTTTTCTCTTGTGGATCCGTATTTTCATAATCATGTTCATAGTAC	cloning	pD_ARK2_icKD
PCR4_ARK2_F	CTTGGATTAAGCAGTTTATTGGAAATAGGTAAATAAAAAAAATAATATAC	cloning	pD_ARK2_icKD
PCR4_ARK2_R	TCTTTTATATGAATTGTTTTTCATTCCAGTTTTAGAAGCTCC	cloning	pD_ARK2_icKD
PCR2_ARK3_F	AGCGAGTCAGTGAGCGAGGATATAAAGCAAAACAATAATACTAAGG	cloning	pD_ARK3_icKD
PCR2_ARK3_R	TTTTTTATTTACCTATTTCCAGTAGTTTTGTTAAGTATAGTTGCTG	cloning	pD_ARK3_icKD
PCR3_ARK3_F	AGCTTCTAAAACTGGAATGACAGAGAAAAATTAAATGGCAACATC	cloning	pD_ARK3_icKD
PCR3_ARK3_R	CCTTTTCTCTTGTGGATCCGTGTTATAATATTCTGATTCATTCCAC	cloning	pD_ARK3_icKD
PCR4_ARK3_F	CTATACTTAACAAAACTACTGGAAATAGGTAAATAAAAAAAATAATATAC	cloning	pD_ARK3_icKD
PCR4_ARK3_R	TGCCATTTAATTTTTCTCTGTCATTCCAGTTTTAGAAGCTCC	cloning	pD_ARK3_icKD
gRNA_ARK1_a	TATTAAACCATATGGGTGTTAGAA	cloning	pHF-gC_ARK1
gRNA_ARK1_b	AAACTTCTAACACCCATATGGTTT	cloning	pHF-gC_ARK1
gRNA_ARK2_a	TATTAACTGCTGAAGAAAGAATTT	cloning	pHF-gC_ARK2
gRNA_ARK2_b	AAACAAATTCTTTCTTCAGCAGTT	cloning	pHF-gC_ARK2
gRNA_ARK3_a	TATTATAAATAAACAGCAAAGATT	cloning	pHF-gC_ARK3
gRNA_ARK3_b	AAACAATCTTTGCTGTTTATTTAT	cloning	pHF-gC_ARK3
